# RNA helicase DDX5 acts as a critical regulator for survival of neonatal mouse gonocytes

**DOI:** 10.1111/cpr.13000

**Published:** 2021-03-05

**Authors:** Qing Xia, Guizhong Cui, Ye Fan, Xiuqin Wang, Gongcheng Hu, Lisha Wang, Xi Luo, Lele Yang, Qingqing Cai, Kaibiao Xu, Wenjing Guo, Minghui Gao, Yingying Li, Ji Wu, Wei Li, Jiayu Chen, Huayu Qi, Guangdun Peng, Hongjie Yao

**Affiliations:** ^1^ CAS Key Laboratory of Regenerative Biology and Guangdong Provincial Key Laboratory of Stem Cell and Regenerative Medicine Joint School of Life Sciences Guangzhou Institutes of Biomedicine and Health Chinese Academy of Sciences Guangzhou Medical University Guangzhou China; ^2^ GIBH‐HKU Guangdong‐Hong Kong Stem Cell and Regenerative Medicine Research Centre Guangzhou Institutes of Biomedicine and Health Chinese Academy of Sciences Guangzhou China; ^3^ Bioland Laboratory (Guangzhou Regenerative Medicine and Health GuangDong Laboratory) Guangzhou China; ^4^ Institute of Stem Cell and Regeneration Chinese Academy of Sciences Beijing China; ^5^ University of Chinese Academy of Sciences Beijing China; ^6^ Department of Respiratory Disease Xinqiao Hospital Third Military Medical University Chongqing China; ^7^ Bio‐X Institutes Shanghai Jiao Tong University Shanghai China; ^8^ State Key Laboratory of Stem Cell and Reproductive Biology Institute of Zoology Chinese Academy of Sciences Beijing China; ^9^ Clinical and Translation Research Center of Shanghai First Maternity and Infant Hospital Shanghai Key Laboratory of Signaling and Disease Research School of Life Sciences and Technology Tongji University Shanghai China

**Keywords:** DDX5, gonocyte, RNA‐binding protein, spermatogonial stem cell, testis

## Abstract

**Objectives:**

Mammalian spermatogenesis is a biological process of male gamete formation. Gonocytes are the only precursors of spermatogonial stem cells (SSCs) which develop into mature spermatozoa. DDX5 is one of DEAD‐box RNA helicases and expresses in male germ cells, suggesting that *Ddx5* plays important functions during spermatogenesis. Here, we explore the functions of *Ddx5* in regulating the specification of gonocytes.

**Materials and Methods:**

Germ cell‐specific *Ddx5* knockout (*Ddx5* ^‐/‐^) mice were generated. The morphology of testes and epididymides and fertility in both wild‐type and *Ddx5* ^‐/‐^ mice were analysed. Single‐cell RNA sequencing (scRNA‐seq) was used to profile the transcriptome in testes from wild‐type and *Ddx5* ^‐/‐^ mice at postnatal day (P) 2. Dysregulated genes were validated by single‐cell qRT‐PCR and immunofluorescent staining.

**Results:**

In male mice, *Ddx5* was expressed in germ cells at different stages of development. Germ cell‐specific *Ddx5* knockout adult male mice were sterile due to completely devoid of germ cells. Male germ cells gradually disappeared in *Ddx5* ^‐/‐^ mice from E18.5 to P6. Single‐cell transcriptome analysis showed that genes involved in cell cycle and glial cell line‐derived neurotrophic factor (GDNF) pathway were significantly decreased in *Ddx5*‐deficient gonocytes. Notably, *Ddx5* ablation impeded the proliferation of gonocytes.

**Conclusions:**

Our study reveals the critical roles of *Ddx5* in fate determination of gonocytes, offering a novel insight into the pathogenesis of male sterility.

## Graphical abstract

Under normal physiologic conditions in the neonatal testes, exogenous growth factor GDNF stimulates intracellular signaling through binding to its receptor GFRα1 and RET in cell membranes of the gonocytes. Subsequently, spermatogenesis is believed to initiate shortly after gonocytes give rise to spermatogonia. Spermatogonia could proliferate via mitosis and differentiate to produce spermatocytes. Germ cell‐specific knockout of *Ddx5* results in low expression level of GDNF signaling pathway‐related genes (such as receptor *Gfra1* and *Ret*) in gonocytes. The disruption of spermatogenesis takes place before spermatogonia formation in the testes of *Ddx5* konckout mice. *Ddx5* deletion leads to a complete lack of germ cells and infertility in male adults.

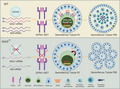

## 1 INTRODUCTION

In mice, as the foundation for the continuity of spermatogenesis, SSCs are derived from gonocytes that are originated from primordial germ cells (PGCs) residing in the proximal epiblast during gastrulation.^1^, ^2^, ^3^ PGCs migrate to the developing gonad and differentiate into male or female germ cells following sex determination.^4^, ^5^, ^6^, ^7^, ^8^ In the fetal male gonads, germ cells are referred to as gonocytes.^9^ Testicular gonocytes stay in the G0/G1 phase of cell cycle from embryonic day (E) 13.5.^10^, ^11^ The quiescent gonocytes begin to relocate to the periphery from the central region of seminiferous tubules, and resume mitotic division shortly after birth.^10^, ^12^ Gonocytes give rise to undifferentiated spermatogonia or differentiating spermatogonia through gonocytes‐to‐spermatogonia transition (GST), which is the gateway to spermatogenesis in neonatal testes. Proliferation and migration of gonocytes are two crucial events that take place between P0 and P6.^13^, ^14^, ^15^, ^16^ Gonocytes and spermatogonia are closely related, but they have different morphology, transcriptome, DNA methylation and chromosome architecture.^12^, ^15^

Although tremendous efforts have been focused on studying the maintenance and differentiation of SSCs, the formation of SSCs (especially the proliferation, migration and transition of gonocytes), is still unclear. Sertoli cells have been reported to play important roles in regulating these processes. Platelet‐derived growth factor (PDGF), synthesized and secreted by Sertoli cells, has the remarkable capability to drive the survival and migration of the gonocytes.^17^, ^18^ As a RNA‐binding protein, DDX5 has been shown to be involved in multiple biological processes. *Ddx5* has been reported to express in male germline^19^, ^20^, ^21^ and plays a negative role in WNT signaling regulation of the GC‐1 spg cell.^22^ Disruption of *Ddx5* in adult spermatogonia results in infertility.^23^ Although it is clear that *Ddx5* is required for the maintenance of spermatogonia in adult testis, we do not yet know whether it is required for the formation of the spermatogonia.

In this study, we found that *Ddx5* was highly expressed in male germ cells from prenatal period. We generated germ cell‐specific *Ddx5* knockout mice by using transgenic mice expressing Cre recombinase driven by the *Mvh* promoter (*Mvh*‐Cre) and found that *Ddx5* ^‐/‐^ mice showed significantly smaller and lighter testes than wild‐type (WT) and heterozygous (*Ddx5* ^+/‐^) mice. Furthermore, *Ddx5* knockout resulted in a complete loss of germ cells, containing only Sertoli cells within 6 days after birth, and led to azoospermia and infertility in male adults. In addition, scRNA‐seq experiments indicated that *Ddx5* knockout resulted in aberrant expression of genes that were associated with cell cycle and GDNF pathway in gonocytes. Interestingly, we further found that *Ddx5* knockout induced dysregulation of GDNF signaling pathway and then impeded proliferation of gonocytes and gonocytes‐to‐spermatogonia transition.

## 2 MATERIALS AND METHODS

### 2.1 Generation of *Ddx5* conditional knockout mice

The *Ddx5* floxed mice^24^ *Ddx5* ^flox/flox^ were crossed with *Mvh*‐Cre transgenic mice^25^ to obtain *Ddx5* ^+/‐^ mice. Homozygous *Ddx5* ^‐/‐^ mice were obtained by crossing *Ddx5* ^+/‐^ males and *Ddx5* ^flox/flox^ females.

### 2.2 Assessment of fertility and fecundity

To assess fertility and fecundity, one 3‐month‐old male mouse of either wild‐type, *Ddx5* ^+/‐^ or *Ddx5* ^‐/‐^ was placed into a cage with a wild‐type female. Cages were monitored daily. The females were checked for the presence of vaginal plugs and pregnancy. The numbers of pups born in each mating set were recorded.

### 2.3 Immunofluorescent staining of the frozen sections

Tissues were dissected from mice immediately after euthanasia and fixed in 4% paraformaldehyde (PFA) (Beyotime, P0099) overnight at 4°C. Samples were embedded with tissue freezing medium (Leica, 020108926) and sectioned (8 μm in thickness). The sections were permeabilized with 0.3% Triton X‐100 (Sigma‐Aldrich, T8787) in PBS for 20 minutes and blocked with 5% BSA (Gold Bio, A‐420) in PBS for 1 hour at room temperature. The sections were incubated with the diluted primary antibody and subsequently secondary antibody. Primary antibodies used in this study are listed in Table S2. Immunoglobulin G (IgG) was used as a negative control for the primary antibody. A final concentration of 1 μg/mL DAPI (Beyotime, C1002) was included to stain nuclei. Fluorescent images were captured with a confocal microscope (LSM800, Carl Zeiss). Images were further processed with ZEN‐2012SP2‐blue software. Numbers of gonocytes and the area corresponding to testis cross‐section were measured using ImageJ software. To minimize the difference caused by different testicular positions, the gonocytes were shown as unit area (mm^2^).

### 2.4 RNA extraction and quantitative RT‐PCR (qRT‐PCR)

Total RNA was extracted from mouse testes using TRIzol reagent (MRC, TR118) in accordance with the manufacturer's instructions. Nucleic acid quantification and purity are listed in Table S4. Briefly, 1 μg of total RNA was reverse‐transcribed with HiScript II Q RT SuperMix for qPCR with gDNA Wiper (Vazyme, R223‐01). Quantitative RT‐PCR was performed using a CFX96 Real‐Time System (Bio‐Rad) and SYBR Green qPCR Mix (GenStar, A301‐01). The PCR cycling conditions were as follows: 40 cycles of 95°C for 10 seconds (denaturation), 60°C for 10 seconds (annealing) and 72°C for 20 seconds (elongation). Expression levels were normalized to the geometric mean of *Gapdh* and *Actin*. The relative expression level of candidate genes was calculated using the formula 2^‐ΔΔCT^ as described.^26^ The primers used are listed in Table S1. All experiments were repeated three times.

### 2.5 Isolation of single testicular cells by fluorescence‐activated cell sorting (FACS), single‐cell reverse transcription and amplification

The testes at P2 were collected, de‐capsulated and digested with TrypLE™ Express Enzyme (Thermo Fisher, 12605010). The cell suspensions were filtered through a 40 μm cell strainer (Corning, 22363547), and cells were collected by centrifugation at 300 × *g* for 5 minutes. The pellets were resuspended in DPBS (HyClone, SH30028) with 0.04% BSA and isolated with a MoFlo Astrios EQ high speed cell sorter (Beckman Coulter). Then, the cells were sorted into cell lysis buffer containing 0.45% (vol/vol) NP40 (Roche, 11332473001), followed by reverse transcription using SuperScript II Reverse Transcriptase (Invitrogen, 18064‐014) and whole transcription amplification using KAPA HiFi HotStart Ready Mix (KAPA Biosystems, KK2602).^27^

### 2.6 10 × genomics single‐cell sample processing and cDNA library preparation

Single testicular cells of testes at P2 were dissociated with TrypLE™ Express Enzyme (Thermo Fisher, 12605010). Cell viability was assessed by trypan blue staining. Cell capturing, cDNA amplification and library preparation were carried by Chromium Single Cell 3’ GEM, Library & Gel Bead Kit v3 (10 × Genomics, 1000075) according to the manufacturer's instructions. The resultant libraries were size selected, pooled and sequenced using 2 × 150 paired‐end sequencing protocol on an Illumina NovaSeq 6000 instrument.

## 3 RESULTS

### 3.1 *Ddx5* expression during testicular development

**FIGURE 1:**
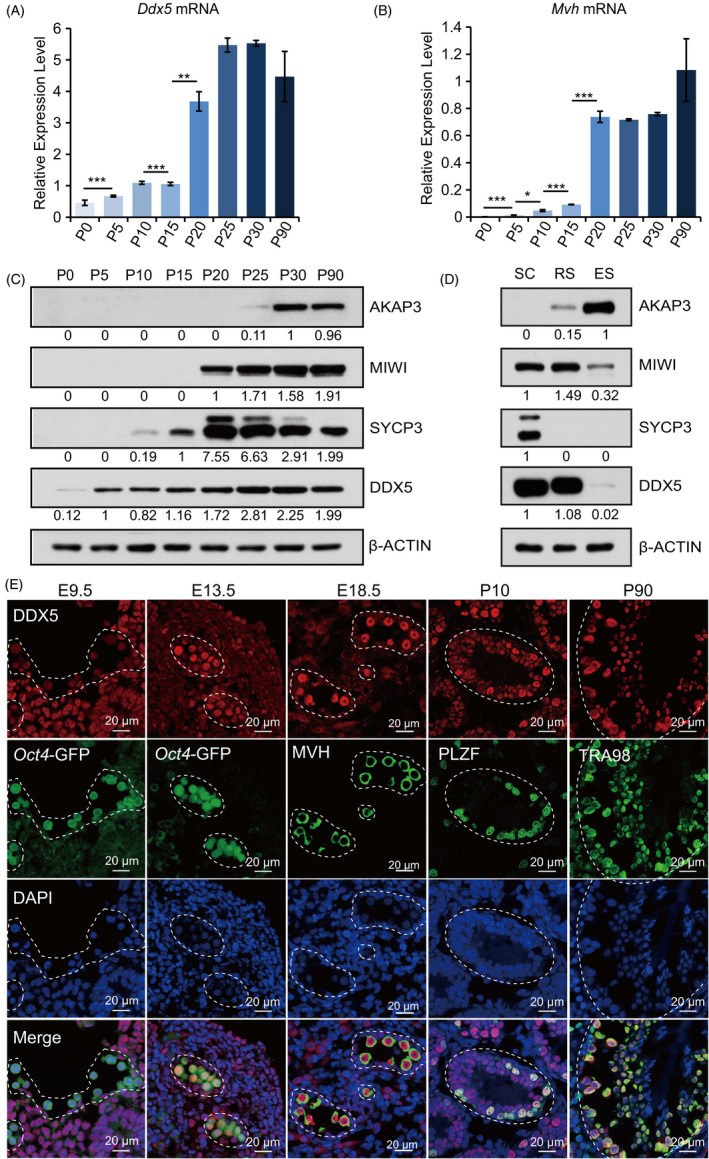
Expression analysis of *Ddx5* during development of male germline. A and B, qRT‐PCR to analyse the expression of *Ddx5* and germ cell marker *Mvh* in the testes of mice at different stages (P0 to P90). Expression levels were normalized against geometric mean of *Gapdh* and *Actin*. Error bars correspond to means ± SD (*P < .05, **P < .01, ***P < .001). C, Western blotting to analyse DDX5 protein in the testes of mice at different stages (P0 to P90). AKAP3 was used as elongating spermatid marker. MIWI was used as spermatocyte and round spermatid marker. SYCP3 was used as spermatocytes marker. β‐ACTIN was used as a loading control. D, Western blotting to analyse protein level of AKAP3, MIWI, SYCP3 and DDX5 in the fractionated testicular spermatocytes (SC), round spermatids (RS) and elongating spermatids (ES). β‐ACTIN was used as a loading control. E, Immunofluorescent staining of DDX5 expression in germ cells from wild‐type mice at E9.5 to P90. Germ cells were labelled with *Oct4*‐GFP (green) in E9.5 and E13.5 gonad sections. Spermatogonia were labelled with anti‐PLZF antibody in testis section at P10. All germ cells except elongating spermatids were labelled with anti‐TRA98 antibody in testis section at P90. DNA was stained with DAPI (blue). Scale bars represent 20 μm

To explore the roles of *Ddx5* during male germ cell development, *Ddx5* expression was evaluated in testes of postnatal mice, collecting every 5 days after birth according to the developmental process of spermatogenic cells.^28^ The mRNA level of *Ddx5* was increased accompanying mouse age, with more prominent expression at P20 and P25, similar to that of *Mvh,* a germ cell‐specific marker (Figure 1A,B). Moreover, we performed western blotting for DDX5 and different markers of germ cells, respectively. As expected, DDX5 protein level was gradually increased from P0 to P90, and increasing levels as spermatocytes and round spermatids developed (Figure 1C). Western blotting of spermatogenic cells isolated using unit‐gravity sedimentation indicated that DDX5 was predominantly expressed in spermatocytes and round spermatids, but not in elongating spermatids (Figure 1D).

We further examined the expression and subcellular localization of DDX5 during earlier male germ lineage development by performing immunofluorescent staining (Figure 1E). Transgenic mice which express the *Oct4* promoter‐driven GFP (hereafter referred to as *Oct4*‐GFP) were used to mark germ cells. Our data indicated that DDX5 had lower level in PGCs at E9.5, but prominently expressed in gonocytes at E13.5 and E18.5 following sexual differentiation, as well as in postnatal germ cells (Figure 1E). Moreover, we observed that DDX5 expression was relatively weak in somatic cells from embryonic period. DDX5 was expressed in the nucleus. Together, these data indicate that DDX5 is not only abundant in the nucleus of germ cells after birth, but also in gonocytes in prenatal period, suggesting that *Ddx5* may regulate fate determination of germ cells at embryonic stages.

### 3.2 Germ cell‐specific *Ddx5* knockout leads to infertility

**FIGURE 2:**
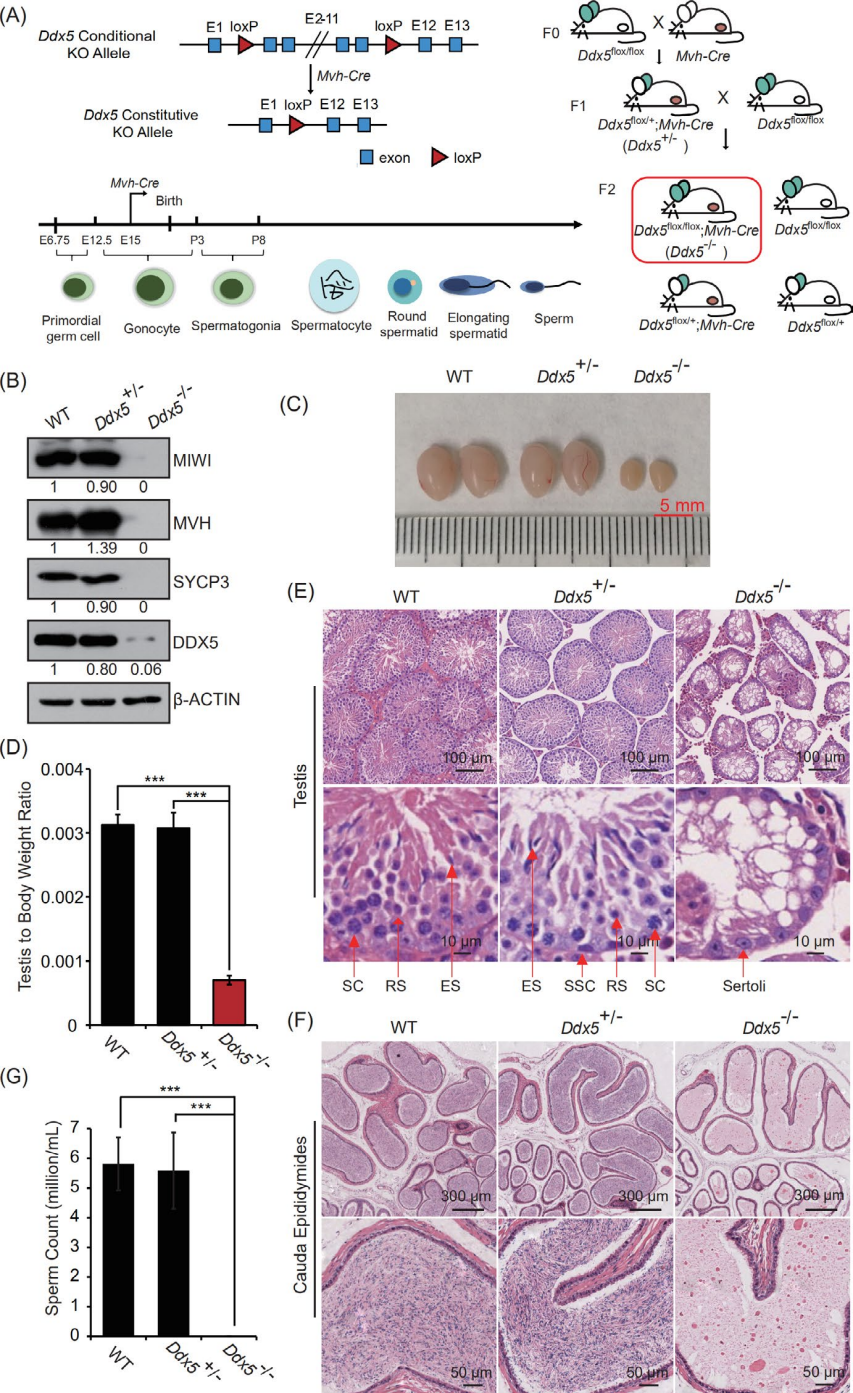
*Ddx5* is essential for spermatogenesis. A, Schematic diagram of *Ddx5* conditional knockout strategy. Left panel, the *Ddx5* allele is flanked by two loxP sites. The blue squares stand for *Ddx5* exon, and the red triangles represent loxP site. Right panel, reproductive strategy for *Ddx5* ^‐/‐^ mice. B, Western blotting to detect protein level of DDX5, MIWI, MVH and SYCP3 in the testes of wild‐type (WT), *Ddx5* ^+/‐^ and *Ddx5* ^‐/‐^ adult mice. β‐ACTIN was used as a loading control. C, Morphological analysis of testes in WT, *Ddx5* ^+/‐^ and *Ddx5* ^‐/‐^ adult mice. Scale bar represents 5 mm. D, Assessment of testis to body weight ratio (mg/mg) from WT, *Ddx5* ^+/‐^ and *Ddx5* ^‐/‐^ adult male mice. Error bars correspond to means ± SD. *P* = .4428 between WT and *Ddx5* ^+/‐^, *P* = 3.644e‐05 between *Ddx5* ^+/‐^ and *Ddx5* ^‐/‐^ and *P* = 7.396e‐07 between WT and *Ddx5* ^‐/‐^ (***P < .001, n = 6). E, H&E staining of testis sections from WT, *Ddx5* ^+/‐^ and *Ddx5* ^‐/‐^ adult mice. The gaps between the seminiferous tubules due to the processing of testis section preparation. Scale bars represent 100 μm and 10 μm, respectively. SC, spermatocytes, ES, elongating spermatids, RS, round spermatids, SSC, spermatogonial stem cells, Sertoli, Sertoli cells. F, H&E staining of cauda epididymis sections from WT, *Ddx5* ^+/‐^ and *Ddx5* ^‐/‐^ adult male mice. Scale bars represent 300 μm and 50 μm, respectively. G, Quantification of sperms released from cauda epididymides of WT, *Ddx5* ^+/‐^ and *Ddx5* ^‐/‐^ adult males. Error bars correspond to means ± SD. *P* = .6646 between WT and *Ddx5* ^+/‐^, *P* = 1.027e‐05 between *Ddx5* ^+/‐^ and *Ddx5* ^‐/‐^, and *P* = 1.467e‐10 between WT and *Ddx5* ^‐/‐^ (***P < .001, n = 6)

DDX5 is a multi‐functional protein and is involved in several cellular processes.^29^, ^30^ To further explore the role of *Ddx5* in male germline development, *Ddx5* ^‐/‐^ mice were generated by breeding *Ddx5* floxed mice (*Ddx5* ^flox/flox^) with *Mvh*‐Cre mice (Figure 2A). *Ddx5* ^+/‐^ and *Ddx5* ^‐/‐^ mice were born followed Mendel's laws of inheritance and appeared healthy (Figure S1A). Western blotting showed that DDX5 protein was expressed in the testes of both wild‐type and *Ddx5* ^+/‐^ adult mice but was faint in the testes of *Ddx5* ^‐/‐^ adult mice (Figure 2B and Figure S1C). And strikingly, no pups were born when *Ddx5* ^‐/‐^ male mice mated with wild‐type female mice, even copulatory plugs were routinely observed (Table 1). Together, these data reveal that germ cell‐specific knockout of *Ddx5* leads to male infertility.

**TABLE 1:** The fertility of *Ddx5* conditional knockout male mice

Genotype		NO^1^. of male mice	NO. of plugged female mice	NO. of litters	NO. of pups per litter
Male	Female				
WT^2^	WT	7	24	184	7.67
*Ddx5* ^+/‐3^	WT	7	24	175	7.29
*Ddx5* ^‐/‐4^	WT	7	24	0	0

Note

1. Number. 2. Wild‐type. 3. Heterozygotes *Ddx5^+/‐^*. 4. Homozygotes *Ddx5* ^‐/‐^.

### 3.3 *Ddx5* deletion results in the depletion of germ cells in adult male mice

To assess the reason of infertility in *Ddx5*‐deficient male mice, we observed that the testes of *Ddx5* ^‐/‐^ mice were significantly smaller and lighter than those of wild‐type and *Ddx5* ^+/‐^ mice (Figure 2C,D). Haematoxylin and eosin (H&E) staining showed that although the architecture of seminiferous tubules largely preserved, all tubules were atrophy and empty in the testes of *Ddx5* ^‐/‐^ mice compared with multiple layers spermatogenic cells in the testes of wild‐type and *Ddx5* ^+/‐^ mice (Figure 2E). In addition, *Ddx5* ^‐/‐^ mice resulted in loss of mature sperms in the cauda epididymides (Figure 2F,G). Consistently, germ cell‐specific markers were not detectable in *Ddx5* ^‐/‐^ mice (Figure 2B and Figure S1C). Meanwhile, flow cytometry analysis identified stage‐specific subpopulations of spermatogenic cells in wild‐type, but not in *Ddx5* ^‐/‐^ mice (Figure S1B). The above data demonstrate that *Ddx5* ^‐/‐^ adult male mice have no capacity to produce germ cells.

### 3.4 *Ddx5* deletion results in a Sertoli cell‐only phenotype in adult male mice

**FIGURE 3:**
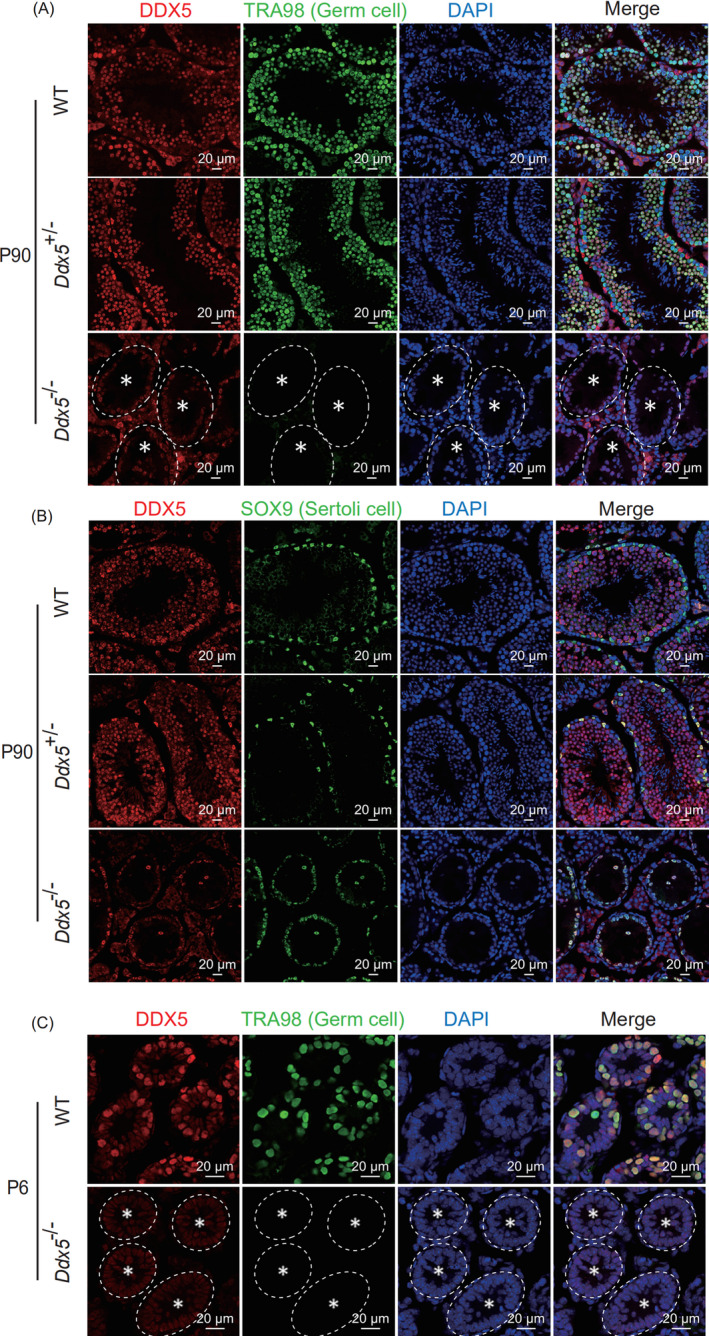
Lack of germ cells in *Ddx5* ^‐/‐^ testes. A, Immunofluorescent staining of DDX5 and TRA98 (germ cells) in testis sections from WT, *Ddx5* ^+/‐^ and *Ddx5* ^‐/‐^ mice at P90. Null tubules were marked with asterisk. B, Immunofluorescent staining of DDX5 and SOX9 (Sertoli cells) in the testes from WT, *Ddx5* ^+/‐^ and *Ddx5* ^‐/‐^ mice at P90. C, Immunofluorescent staining of DDX5 and TRA98 in testis sections from WT and *Ddx5* ^‐/‐^ mice at P6. Null tubules were marked with asterisk. DNA was stained with DAPI. Scale bars represent 20 μm

Although seminiferous tubules were empty in *Ddx5* ^‐/‐^ adult mice, there was still one layer of cells in peripheral regions of seminiferous tubules. It has been reported that Sertoli cells and SSCs are located in the basement membrane.^31^ To examine the identity of the cells remained in seminiferous tubules, we performed immunofluorescent staining and observed that *Ddx5* ^‐/‐^ mice resulted in loss of TRA98‐positive germ cells compared with wild‐type and *Ddx5* ^+/‐^ mice, but only SOX9‐positive Sertoli cells left (Figure 3A,B). The phenotype that *Ddx5* ^‐/‐^ adult male mice were completely devoid of germ cells is consistent with Sertoli cell‐only syndrome associated with human infertility. SSCs could not maintain in *Ddx5* ^‐/‐^ adult mice, we wondered whether *Ddx5* affects the source of SSCs. We compared the testes in wild‐type and *Ddx5* ^‐/‐^ mice at P6. Immunofluorescent staining with TRA98 indicated that SSCs were completely depleted in *Ddx5* ^‐/‐^ seminiferous tubules (Figure 3C). Therefore, these results indicate that *Ddx5* regulates male germline development at the early stage, even before spermatogonia pool is formed at P6.

### 3.5 Germ cells are gradually lost in testes of *Ddx5* ^‐/‐^ neonates

**FIGURE 4:**
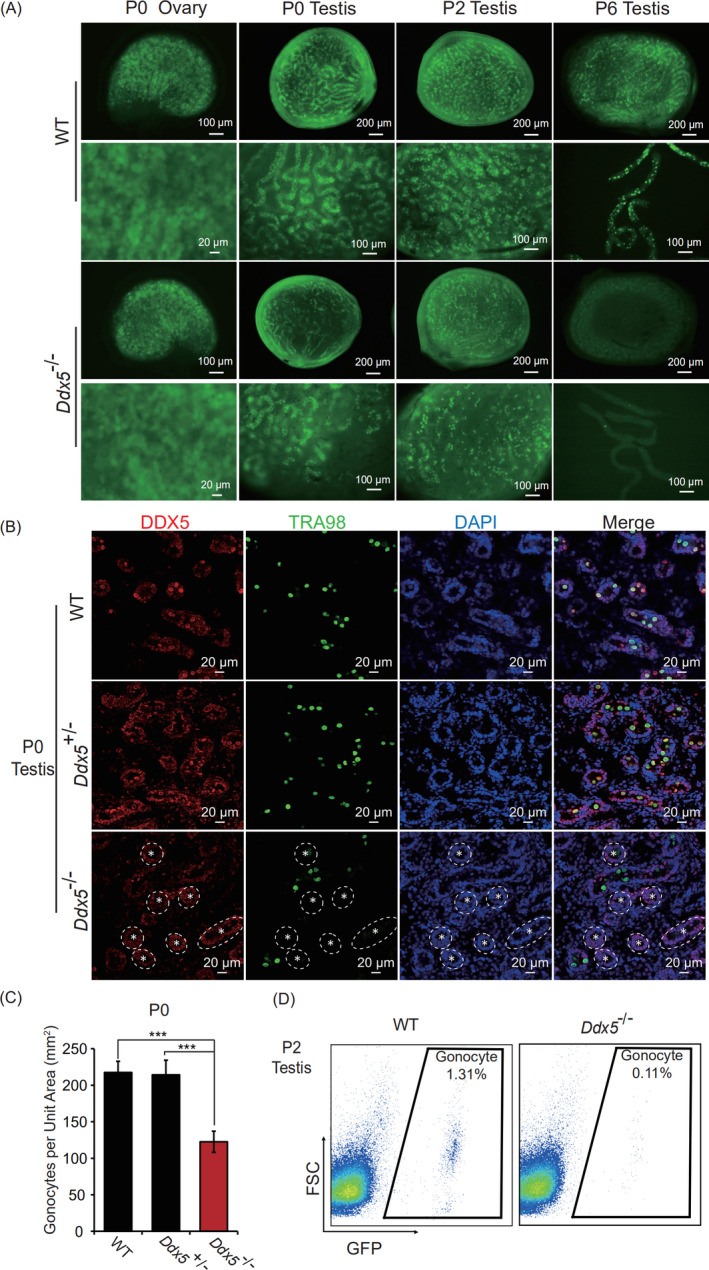
Effects of *Ddx5* knockout on the development of germ cells in neonatal mice. A, *Oct4*‐GFP expression in neonatal testes and ovaries in both WT and *Ddx5* ^‐/‐^ mice. B, Immunofluorescent staining of DDX5 and TRA98 in the testes of WT, *Ddx5* ^+/‐^ and *Ddx5* ^‐/‐^ mice at P0. Null tubules were marked with asterisk. DNA was stained with DAPI. Scale bars represent 20 μm. C, Numbers of TRA98‐positive germ cells (gonocytes) per unit area were counted in the testes of WT, *Ddx5* ^+/‐^ and *Ddx5* ^‐/‐^ male mice at P0. Error bars correspond to means ± SD.*P* = .8534 between WT and *Ddx5* ^+/‐^, *P* = 1.083e‐05 between *Ddx5* ^+/‐^ and *Ddx5* ^‐/‐^, and *P* = 1.083e‐05 between WT and *Ddx5* ^‐/‐^ (***P < .001, n = 10). D, *Oct4*‐GFP‐positive gonocytes were analysed by FACS in testicular cells of WT, *Ddx5* ^+/‐^ and *Ddx5* ^‐/‐^ mice at P2. FSC, forward scatter

*Mvh*‐Cre transgenic mice express recombinase faintly at E15 and strongly at E18. To explore the effect of *Ddx5* on germ cells during the embryonic stage, we collected testes at E18.5 and found that *Ddx5* knockout has no effect on the number of gonocytes (Figure S2A,B). Therefore, we focused on the effect of *Ddx5* loss on postnatal days. We observed that *Oct4*‐GFP‐positive germ cells were reduced in the testes of *Ddx5* ^‐/‐^ mice at P0 and P2, and were totally absent at P6. In contrast, no change was observed in ovaries between wild‐type and *Ddx5* ^‐/‐^ mice (Figure 4A). Additionally, immunofluorescent staining revealed that *Ddx5* ^‐/‐^ mice had significantly reduced numbers of gonocytes in seminiferous tubules at P0 (Figure 4B), and about 42% gonocytes were diminished in the testes of *Ddx5* ^‐/‐^ mice (Figure 4C). We also analysed the percentage of *Oct4*‐GFP‐positive cells using flow cytometry at P2. Noticeably, *Ddx5* ^‐/‐^ mice had only 0.11% gonocytes in all testicular cells while wild‐type mice had 1.31% (Figure 4D). Taken together, these results demonstrate that gonocytes are gradually reduced and then completely depleted from E18.5 to P6 upon *Ddx5* ablation, suggesting that *Ddx5* is indispensable for the survival of gonocytes in postnatal testes.

### 3.6 Single‐cell RNA sequencing analysis reveals the effects of *Ddx5* knockout on gene expression in gonocytes

**FIGURE 5:**
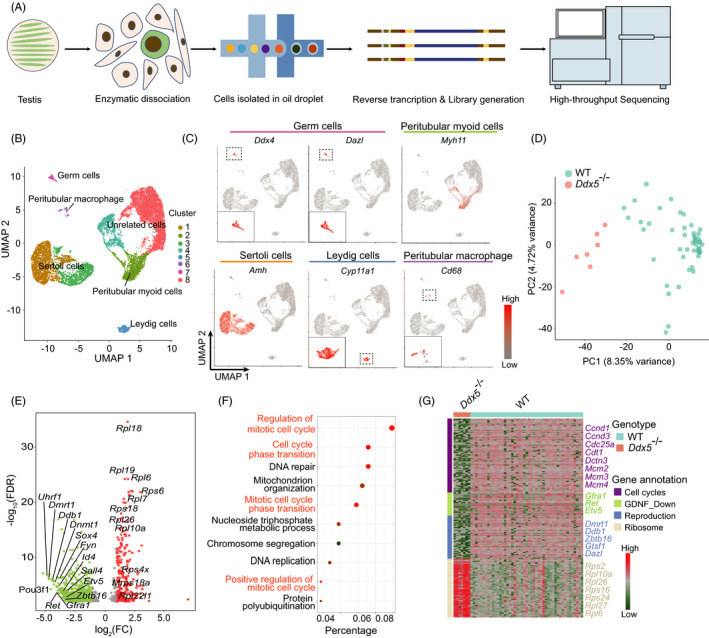
Single‐cell RNA‐seq analysis of testes in both WT and *Ddx5* ^‐/‐^ mice. A, Schematic overview of scRNA‐seq using neonatal mouse testis samples. B, Dimensionality reduction and clustering of testis scRNA‐seq data in WT and *Ddx5* ^‐/‐^ mice at P2 (n = 11 278 cells). Colour coded for clustering analysis groups and annotated post hoc based on their transcriptional profile identities. Signature genes of clusters are listed in Table S3. C, UMAP plots of 5 major cell populations showed the expression of representatively well‐known cell type‐specific marker genes. Gene expression levels are indicated by shades of red. D, PCA plot based on the expression of highly variable genes (n = 3817) in WT (n = 47) and *Ddx5* ^‐/‐^ (n = 7) germ cells. E, Volcano plot displaying DEGs between WT and *Ddx5* ^‐/‐^ germ cells. Genes with a Benjamini‐Hochberg‐adjusted *P* value <.01 and an absolute value of log_2_ fold change (FC) >1 were considered significant. Red represented up‐regulated genes, green represented down‐regulated genes and grey represented non‐critical genes. The most significant DEGs in each direction were labelled. F, GO enrichment showing the terms associated with down‐regulated genes with adjusted *P* value lower than .01 from *Ddx5* ^‐/‐^ germ cells. The dots indicated the GO categories with biological meaning. The colour of dots indicated high (red) or low (dark green) enrichment for a specific GO category. The size of dots displayed the overlap between the input gene lists with the collection of gene sets. G, Heat map showing the expression level of DEGs. The genes were ordered by annotated gene group and representative genes were shown. The colour gradient was row‐normalized across the samples based on log_2_ (TPM + 1)

To investigate the regulatory mechanism of *Ddx5* in gonocytes, scRNA‐seq analysis was performed on dissociated testicular cells, which were isolated from whole testes of wild‐type and *Ddx5* ^‐/‐^ mice at P2 (Figure 5A). We obtained 6328 and 4950 single‐cell transcriptomes in the testes of wild‐type and *Ddx5* ^‐/‐^ mice after quality control, respectively (Figure S3A). Dimensionality reduction and visualization using uniform manifold approximation and projection (UMAP)^32^ allowed us to divide the cells into eight distinct clusters designated as clusters 1 to 8 that can be annotated into germ cells, peritubular macrophages, peritubular myoid cells, Leydig cells and Sertoli cells according to known marker genes (Figure 5B,C; Figure S3B,C; Table S3). The relative proportion of each cluster weighed by the percentage of total cells in both genotypes showed that the ratio of germ cells in the testes was dropped dramatically from 1.86% to 0.24% in *Ddx5* ^‐/‐^ mice compared with wild type, while the other cell clusters remained unchanged (Figure S3D).

To further elucidate the effect of *Ddx5* loss on cellular responses in the testes, germ cells (gonocytes) were selected for further analysis. 54 germ cells (47 from wild type and 7 from *Ddx5* ^‐/‐^) were subjected to principal component analysis (PCA) based on the highly variable genes (n = 3817). We found a segregated distribution for wild‐type and *Ddx5* ^‐/‐^ cells along the PC1 (Figure 5D). The differentially expressed gene analysis identified 848 genes including 232 up‐regulated genes and 616 down‐regulated genes in the gonocytes of *Ddx5* ^‐/‐^ mice (Figure 5E), confirming a profound change in gene expression profiles. Gene Ontology (GO) analysis revealed that the up‐regulated genes were mainly related to the formation of free 40S subunits, ribonucleoprotein complex assembly and mRNA metabolic process (Figure S3E), while the down‐regulated genes were significantly associated with mitotic cell cycle, cell cycle phase transition and DNA repair (Figure 5F,G). Altogether, scRNA‐seq analysis reveals that *Ddx5* knockout obviously disturbs the expression of genes in gonocytes.

### 3.7 *Ddx5* maintains survival of gonocytes through regulating the expression level of genes related to GDNF pathway

**FIGURE 6:**
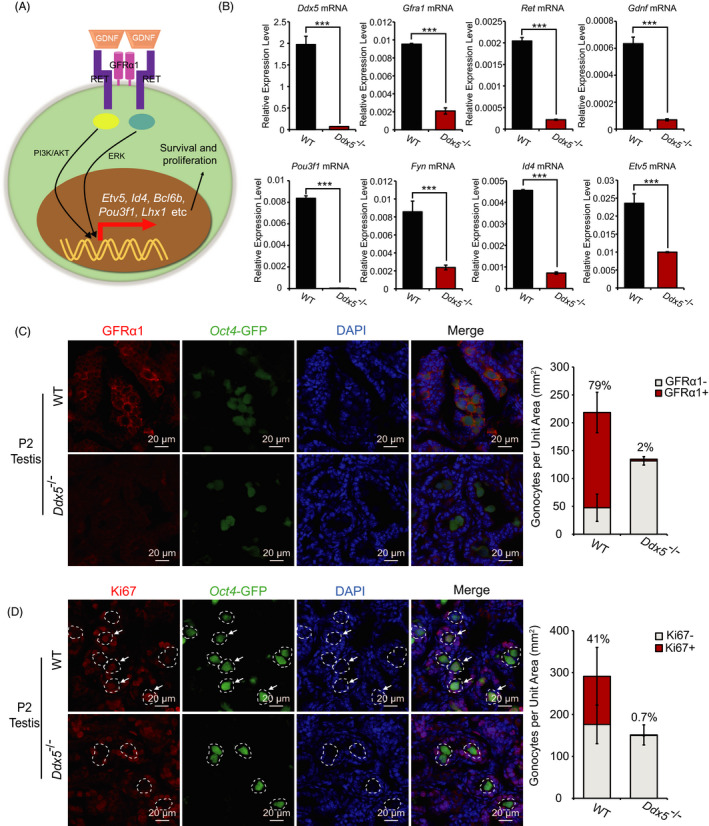
*Ddx5* maintains gonocytes and regulates the transcription of genes in GDNF pathway. A, Schematic diagram showing GDNF‐GFRα1‐RET signaling pathway in the gonocytes. B, Single‐cell qRT‐PCR to analyse the expression of *Ddx5* and GDNF‐GFRα1‐RET signaling pathway‐related genes in *Oct4*‐GFP‐positive gonocytes sorted by FACS in WT and *Ddx5* ^‐/‐^ testicular cells at P2. Expression levels were normalized against geometric mean of *Gapdh* and *Actin*. Error bars correspond to means ± SD (***P < .001, n = 3). C and D, Immunofluorescent staining of GFRα1 and Ki67 in WT and *Ddx5* ^‐/‐^ testes at P2, respectively. Quantification of immunofluorescent signals of GFRα1 and Ki67 per unit area in the testis sections of WT and *Ddx5* ^‐/‐^ male mice at P2, respectively, was calculated in the right panels. The percentage indicates the proportion of GFRα1 or Ki67‐positive gonocytes to all gonocytes in the testis sections. GFRα1‐: GFRα1‐negative gonocytes, GFRα1+: GFRα1‐positive gonocytes, Ki67‐: Ki67‐negative gonocytes, Ki67+: Ki67‐positive gonocytes. Error bars correspond to means ± SD (n = 3). Gonocytes were labelled with *Oct4*‐GFP (green). DNA was stained with DAPI. Scale bars represent 20 μm

After *Ddx5* knockout, the most majority of differentially expressed genes (DEGs) were down‐regulated, and many of them were critical for male germ cell development, including components in GDNF signaling pathway (Figure 5G). GDNF is a growth factor secreted by testicular somatic cells, which is necessary for spermatogonia proliferation and survival.^33^, ^34^, ^35^ GDNF forms a complex with GFRα1, a cell surface receptor of the gonocytes,^36^ and subsequently binds to the transmembrane RET tyrosine kinase and activates intracellular signaling pathways (Figure 6A). Our scRNA‐seq data showed that the GDNF pathway‐related genes were down‐regulated in *Ddx5* ^‐/‐^ male mice (Figure 5G). To confirm the dysregulation of GDNF pathway following *Ddx5* knockout, we performed single‐cell qRT‐PCR analysis on *Oct4*‐GFP‐positive male germ cells, which were isolated from the testes of both wild‐type and *Ddx5* ^‐/‐^ males at P2. Our data showed that the mRNA levels of *Ddx5*, *Gfra1*, *Ret*, *Gdnf*, *Pou3f1*, *Fyn*, *Id4* and *Etv5* were dramatically decreased in *Ddx5*‐deficient gonocytes (Figure 6B), which are consistent with the scRNA‐seq results. Moreover, immunofluorescent staining of cross‐sections showed that GFRα1 protein level was significantly reduced on the surface of gonocytes in *Ddx5* ^‐/‐^ testes compared with that in wild type (Figure 6C). Meanwhile, we barely observed proliferating gonocytes in the newborn *Ddx5* ^‐/‐^ mice by immunofluorescent staining with anti‐Ki67 antibody (Figure 6D). We also examined possible gonocyte apoptosis through TUNEL staining and found that apoptotic cells did not increase in *Ddx5* ^‐/‐^ neonates (Figure S2C). In summary, these data suggest that *Ddx5* knockout disrupts the GDNF signaling pathway and might further lead to phenotypic defects in proliferation and survival of gonocytes.

## 4 DISCUSSION

RNA‐binding protein DDX5 is widely known to participate in various aspects of RNA metabolism.^30^ In this study, we report that *Ddx5* is required for male germ cell development and *Ddx5*‐deficient male mice are infertile due to loss of gonocytes in neonates. The phenotype of *Ddx5* ^‐/‐^ mice is similar to the Sertoli cell–only syndrome (SCO) in humans, which shows the complete absence of germ cells in human testicular tissues.^37^ While the potential mechanism of SCO is still unclear, DDX5 loss might contribute to SCO in humans.

During the process of our study, another independent laboratory reported UBC‐Cre^ERT2^ mediated *Ddx5* knockout affects the maintenance of spermatogonia.^23^ *Ddx5* knockout in spermatogonia at 8 to 12 weeks led to completely devoid of germ cells in seminiferous tubules.^23^ Here, we generated *Ddx5* conditional knockout mice by using *Mvh*‐Cre to delete *Ddx5* from embryonic period and found that the infertility of adult male mice after *Ddx5* loss was due to low survival of gonocytes. Based on theirs and our study, both of us acquired the similar Sertoli cell‐only phenotype and male infertility in adult mice. However, compared with the discoveries from Legrand et al,^23^ we mainly focused on the roles of *Ddx5* in gonocytes at the early stage from E18.5 to P6. Indeed, our data clearly indicated that *Ddx5* was important for the survival and transition of gonocytes. However, it remains unknown how *Ddx5* regulates gene expression of gonocytes. DDX5 is widely known to be involved in several steps of RNA metabolism and ribosome biogenesis,^30^, ^38^, ^39^ in which many genes were dysregulated in *Ddx5* ^‐/‐^ gonocytes. Further, the significantly enriched pathways of down‐regulated genes were cell cycle‐related pathways, which were consistent with the findings in spermatogonia.^23^ Moreover, many genes involved in male germ cell development were significantly down‐regulated in gonocytes of *Ddx5* ^‐/‐^ mice, including almost all of the genes related to GDNF‐GFRα1‐RET signaling pathway (eg, *Gfra1*, *Ret*, *Etv5* and *Id4*) and spermatogenesis‐related genes (eg, *Zbtb16*, *Sall4*, *Gtsf1* and *Dmrt1*). Among them, ZBTB16 and SALL4 are two important transcription factors for self‐renewal and differentiation of spermatogonia.^40^, ^41^, ^42^, ^43^ In addition, both *Gtsf1* and *Dmrt1* are vital for spermatogenesis beyond the early meiotic phase.^44^, ^45^, ^46^, ^47^ Therefore, additional mechanisms for *Ddx5* in regulating survival of neonatal mouse gonocytes could be pursued by investigating how *Ddx5* modulates the expression of *Zbtb16*, *Sall4*, *Gtsf1* and *Dmrt1*.

In conclusion, the findings presented here demonstrate that *Ddx5* is required and essential for male fertility. Furthermore, *Ddx5* is indispensable for survival and transition of gonocytes in male neonates at least through regulating the genes that associated with cell cycle and GDNF pathway.

## CONFLICT OF INTEREST

The authors declare no competing interests.

## AUTHOR CONTRIBUTIONS

HY and QX initiated the study and designed the experiments. QX conducted most of the experiments. GC performed the bioinformatics analysis. YF provided the *Ddx5* floxed mice. XW, GH, LW, XL, LY, QC, KX, WG, MG and YL provided necessary assistances. JW and WL provided *Mvh*‐Cre transgenic mice. JC and HQ contributed to the work. HY, QX and GC wrote the manuscript. HY and GP conceived and supervised the entire study. HY approved the final version.

## Supporting information

Fig S1

Fig S2

Fig S3

Supplementary Material

Table S1‐S4

## Data Availability

RNA‐seq data generated in this study are deposited in the NCBI Gene Expression Omnibus under accession number GSE158285.

## References

[1] Saitou M , Barton SC , Surani MA . A molecular programme for the specification of germ cell fate in mice. Nature. 2002;418:293‐300. 12124616 10.1038/nature00927

[2] Saitou M . Specification of the germ cell lineage in mice. Front Biosci (Landmark Ed). 2009;14:1068‐1087. 19273116 10.2741/3294

[3] Gunesdogan U , Magnusdottir E , Surani MA . Primordial germ cell specification: a context‐dependent cellular differentiation event [corrected]. Philos Trans R Soc Lond B Biol Sci. 2014;369:20130543. 25349452 10.1098/rstb.2013.0543 PMC4216466

[4] Anderson R , Copeland TK , Scholer H , et al. The onset of germ cell migration in the mouse embryo. Mech Dev. 2000;91:61‐68. 10704831 10.1016/s0925-4773(99)00271-3

[5] Ginsburg M , Snow MH , McLaren A . Primordial germ cells in the mouse embryo during gastrulation. Development. 1990;110:521‐528. 2133553 10.1242/dev.110.2.521

[6] McLaren A . Primordial germ cells in the mouse. Dev Biol. 2003;262:1‐15. 14512014 10.1016/s0012-1606(03)00214-8

[7] Harikae K , Miura K , Kanai Y . Early gonadogenesis in mammals: significance of long and narrow gonadal structure. Dev Dyn. 2013;242:330‐338. 22987627 10.1002/dvdy.23872

[8] Barton LJ , LeBlanc MG , Lehmann R . Finding their way: themes in germ cell migration. Curr Opin Cell Biol. 2016;42:128‐137. 27484857 10.1016/j.ceb.2016.07.007 PMC5064876

[9] Culty M . Gonocytes, the forgotten cells of the germ cell lineage. Birth Defects Res C Embryo Today. 2009;87:1‐26. 19306346 10.1002/bdrc.20142

[10] Nagano R , Tabata S , Nakanishi Y , et al. Reproliferation and relocation of mouse male germ cells (gonocytes) during prespermatogenesis. Anat Rec. 2000;258:210‐220. 10645968 10.1002/(SICI)1097-0185(20000201)258:2<210::AID-AR10>3.0.CO;2-X

[11] Western PS , Miles DC , van den Bergen JA , et al. Dynamic regulation of mitotic arrest in fetal male germ cells. Stem Cells. 2008;26:339‐347. 18024419 10.1634/stemcells.2007-0622

[12] Yamanaka S , Nishihara H , Toh H , et al. Broad Heterochromatic Domains Open in Gonocyte Development Prior to De Novo DNA Methylation. Dev Cell. 2019;51:21‐34.e5. 31474564 10.1016/j.devcel.2019.07.023

[13] Kluin PM , de Rooij DG . A comparison between the morphology and cell kinetics of gonocytes and adult type undifferentiated spermatogonia in the mouse. Int J Androl. 1981;4:475‐493. 7298230 10.1111/j.1365-2605.1981.tb00732.x

[14] Yoshida S , Sukeno M , Nakagawa T , et al. The first round of mouse spermatogenesis is a distinctive program that lacks the self‐renewing spermatogonia stage. Development. 2006;133:1495‐1505. 16540512 10.1242/dev.02316

[15] Tan K , Song HW , Wilkinson MF . Single‐cell RNAseq analysis of testicular germ and somatic cell development during the perinatal period. Development. 2020;147(3):dev183251. 31964773 10.1242/dev.183251 PMC7033731

[16] McCarrey JR . Toward a more precise and informative nomenclature describing fetal and neonatal male germ cells in rodents. Biol Reprod. 2013;89:47. 23843236 10.1095/biolreprod.113.110502 PMC4076367

[17] Gnessi L , Fabbri A , Spera G . Gonadal peptides as mediators of development and functional control of the testis: an integrated system with hormones and local environment. Endocr Rev. 1997;18:541‐609. 9267764 10.1210/edrv.18.4.0310

[18] Basciani S , De Luca G , Dolci S , et al. Platelet‐derived growth factor receptor beta‐subtype regulates proliferation and migration of gonocytes. Endocrinology. 2008;149:6226‐6235. 18687785 10.1210/en.2008-0349

[19] Neuhaus N , Yoon J , Terwort N , et al. Single‐cell gene expression analysis reveals diversity among human spermatogonia. Mol Hum Reprod. 2017;23:79‐90. 28093458 10.1093/molehr/gaw079

[20] O'Bryan MK , Clark BJ , McLaughlin EA , et al. RBM5 is a male germ cell splicing factor and is required for spermatid differentiation and male fertility. PLoS Genet. 2013;9:e1003628. 23935508 10.1371/journal.pgen.1003628 PMC3723494

[21] Zhang H , Wang G , Liu L , et al. KH‐type splicing regulatory protein is a new component of chromatoid body. Reproduction. 2017;154:723‐733. 28871057 10.1530/REP-17-0169

[22] Arun G , Akhade VS , Donakonda S , et al. mrhl RNA, a long noncoding RNA, negatively regulates Wnt signaling through its protein partner Ddx5/p68 in mouse spermatogonial cells. Mol Cell Biol. 2012;32:3140‐3152. 22665494 10.1128/MCB.00006-12 PMC3434522

[23] Legrand JMD , Chan AL , La HM , et al. DDX5 plays essential transcriptional and post‐transcriptional roles in the maintenance and function of spermatogonia. Nat Commun. 2019;10:2278. 31123254 10.1038/s41467-019-09972-7 PMC6533336

[24] Nicol SM , Bray SE , Black HD , et al. The RNA helicase p68 (DDX5) is selectively required for the induction of p53‐dependent p21 expression and cell‐cycle arrest after DNA damage. Oncogene. 2013;32:3461‐3469. 22986526 10.1038/onc.2012.426 PMC3556166

[25] Gallardo T , Shirley L , John GB , et al. Generation of a germ cell‐specific mouse transgenic Cre line. Vasa‐Cre. Genesis. 2007;45:413‐417. 17551945 10.1002/dvg.20310 PMC2597027

[26] Bustin SA . Why the need for qPCR publication guidelines?–The case for MIQE. Methods. 2010;50:217‐226. 20025972 10.1016/j.ymeth.2009.12.006

[27] Chen J , Suo S , Tam PP , et al. Spatial transcriptomic analysis of cryosectioned tissue samples with Geo‐seq. Nat Protoc. 2017;12:566‐580. 28207000 10.1038/nprot.2017.003

[28] Bellve AR , Cavicchia JC , Millette CF , et al. Spermatogenic cells of the prepuberal mouse. Isolation and morphological characterization. J Cell Biol. 1977;74:68‐85. 874003 10.1083/jcb.74.1.68 PMC2109873

[29] Fuller‐Pace FV , Moore HC . RNA helicases p68 and p72: multifunctional proteins with important implications for cancer development. Future Oncol. 2011;7:239‐251. 21345143 10.2217/fon.11.1

[30] Xing Z , Ma WK , Tran EJ . The DDX5/Dbp2 subfamily of DEAD‐box RNA helicases. Wiley Interdiscip Rev RNA. 2019;10:e1519. 30506978 10.1002/wrna.1519 PMC6560643

[31] Chen SR , Liu YX . Regulation of spermatogonial stem cell self‐renewal and spermatocyte meiosis by Sertoli cell signaling. Reproduction. 2015;149:R159‐R167. 25504872 10.1530/REP-14-0481

[32] Becht E , McInnes L , Healy J , et al. Dimensionality reduction for visualizing single‐cell data using UMAP. Nat Biotechnol. 2019;37(1):38‐44. 10.1038/nbt.4314 30531897

[33] Chen LY , Willis WD , Eddy EM . Targeting the Gdnf Gene in peritubular myoid cells disrupts undifferentiated spermatogonial cell development. Proc Natl Acad Sci USA. 2016;113:1829‐1834. 26831079 10.1073/pnas.1517994113 PMC4763785

[34] Naughton CK , Jain S , Strickland AM , et al. Glial cell‐line derived neurotrophic factor‐mediated RET signaling regulates spermatogonial stem cell fate. Biol Reprod. 2006;74:314‐321. 16237148 10.1095/biolreprod.105.047365

[35] Pui HP , Saga Y . Gonocytes‐to‐spermatogonia transition initiates prior to birth in murine testes and it requires FGF signaling. Mech Dev. 2017;144:125‐139. 28341395 10.1016/j.mod.2017.03.002

[36] Airaksinen MS , Saarma M . The GDNF family: signalling, biological functions and therapeutic value. Nat Rev Neurosci. 2002;3:383‐394. 11988777 10.1038/nrn812

[37] Ramphul K , Mejias SG . Sertoli‐Cell‐Only Syndrome. Treasure Island, FL: StatPearls; 2020.

[38] Saporita AJ , Chang HC , Winkeler CL , et al. RNA helicase DDX5 is a p53‐independent target of ARF that participates in ribosome biogenesis. Cancer Res. 2011;71:6708‐6717. 21937682 10.1158/0008-5472.CAN-11-1472 PMC3206203

[39] Jalal C , Uhlmann‐Schiffler H , Stahl H . Redundant role of DEAD box proteins p68 (Ddx5) and p72/p82 (Ddx17) in ribosome biogenesis and cell proliferation. Nucleic Acids Res. 2007;35:3590‐3601. 17485482 10.1093/nar/gkm058 PMC1920232

[40] Costoya JA , Hobbs RM , Barna M , et al. Essential role of Plzf in maintenance of spermatogonial stem cells. Nat Genet. 2004;36:653‐659. 15156143 10.1038/ng1367

[41] Chan AL , La HM , Legrand JMD , et al. Germline Stem Cell Activity Is Sustained by SALL4‐Dependent Silencing of Distinct Tumor Suppressor Genes. Stem Cell Reports. 2017;9:956‐971. 28867346 10.1016/j.stemcr.2017.08.001 PMC5599261

[42] Hobbs RM , Fagoonee S , Papa A , et al. Functional antagonism between Sall4 and Plzf defines germline progenitors. Cell Stem Cell. 2012;10:284‐298. 22385656 10.1016/j.stem.2012.02.004 PMC3299297

[43] Hobbs RM , Seandel M , Falciatori I , et al. Plzf regulates germline progenitor self‐renewal by opposing mTORC1. Cell. 2010;142:468‐479. 20691905 10.1016/j.cell.2010.06.041 PMC3210556

[44] Yoshimura T , Toyoda S , Kuramochi‐Miyagawa S , et al. Gtsf1/Cue110, a gene encoding a protein with two copies of a CHHC Zn‐finger motif, is involved in spermatogenesis and retrotransposon suppression in murine testes. Dev Biol. 2009;335:216‐227. 19735653 10.1016/j.ydbio.2009.09.003

[45] Raymond CS , Murphy MW , O'Sullivan MG , et al. Dmrt1, a gene related to worm and fly sexual regulators, is required for mammalian testis differentiation. Genes Dev. 2000;14:2587‐2595. 11040213 10.1101/gad.834100 PMC316999

[46] Krentz AD , Murphy MW , Kim S , et al. The DM domain protein DMRT1 is a dose‐sensitive regulator of fetal germ cell proliferation and pluripotency. Proc Natl Acad Sci USA. 2009;106:22323‐22328. 20007774 10.1073/pnas.0905431106 PMC2799724

[47] Fahrioglu U , Murphy MW , Zarkower D , et al. mRNA expression analysis and the molecular basis of neonatal testis defects in Dmrt1 mutant mice. Sex Dev. 2007;1:42‐58. 18391515 10.1159/000096238

