# Supplementary figures and images for: RNA helicase DDX5 acts as a critical regulator for survival of neonatal mouse gonocytes
Source: Cell Prolif. 2021 Mar 5;54(5):e13000. doi: 10.1111/cpr.13000 (PMC8088469; doi:10.1111/cpr.13000)

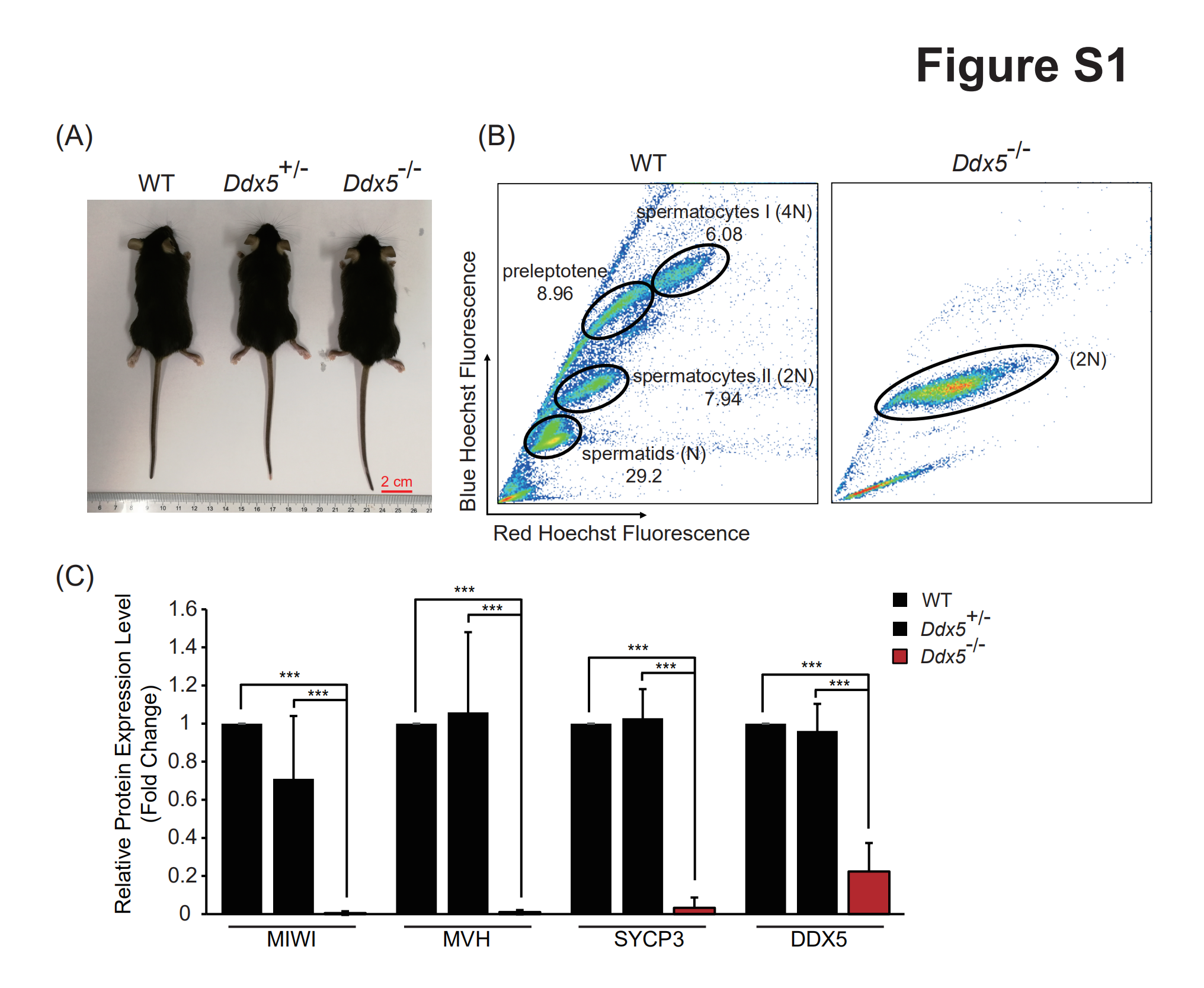

Supplement: Supplementary file 1 — Fig S1 [file CPR-54-e13000-s002.tif]

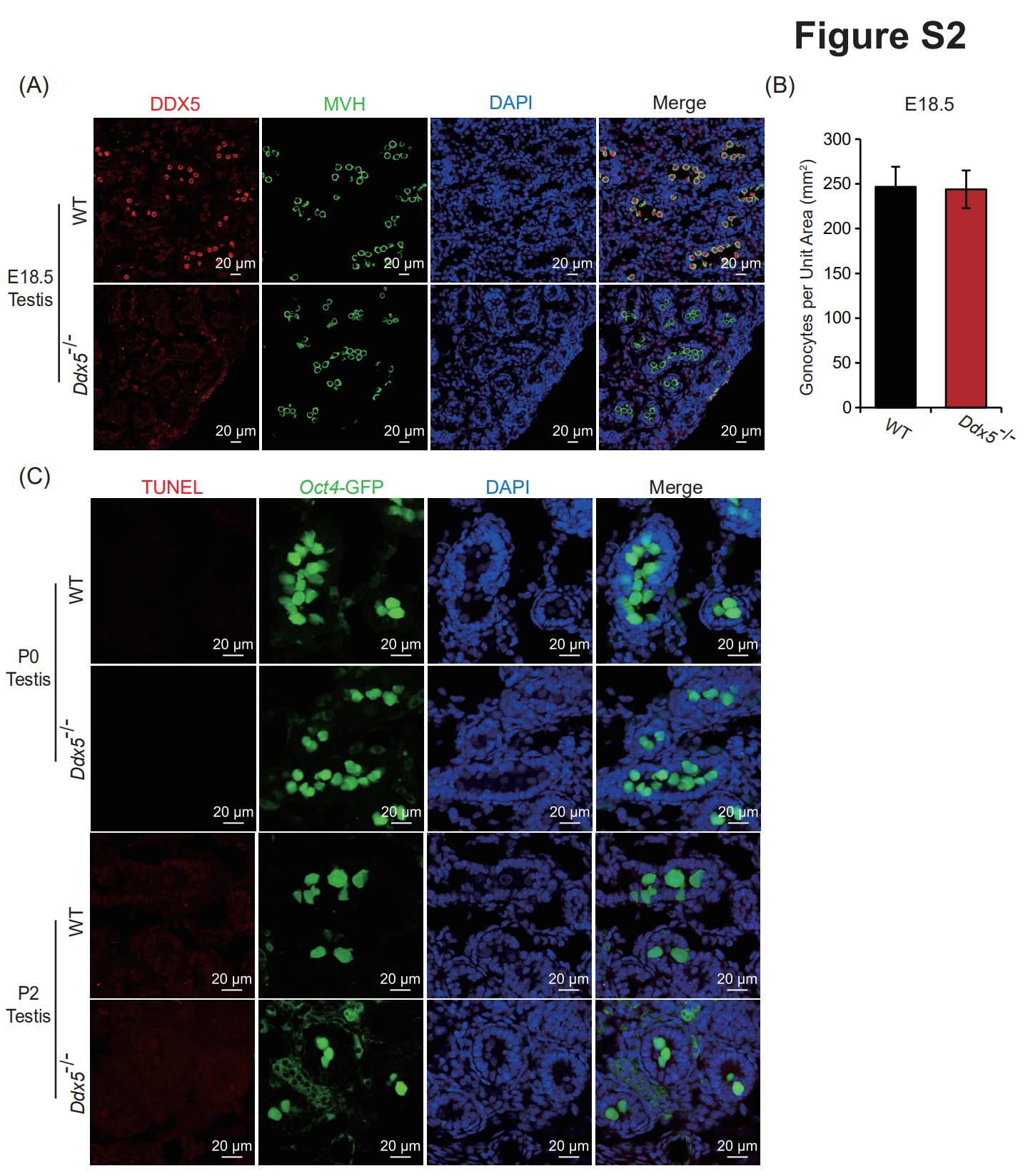

Supplement: Supplementary file 2 — Fig S2 [file CPR-54-e13000-s001.tif]

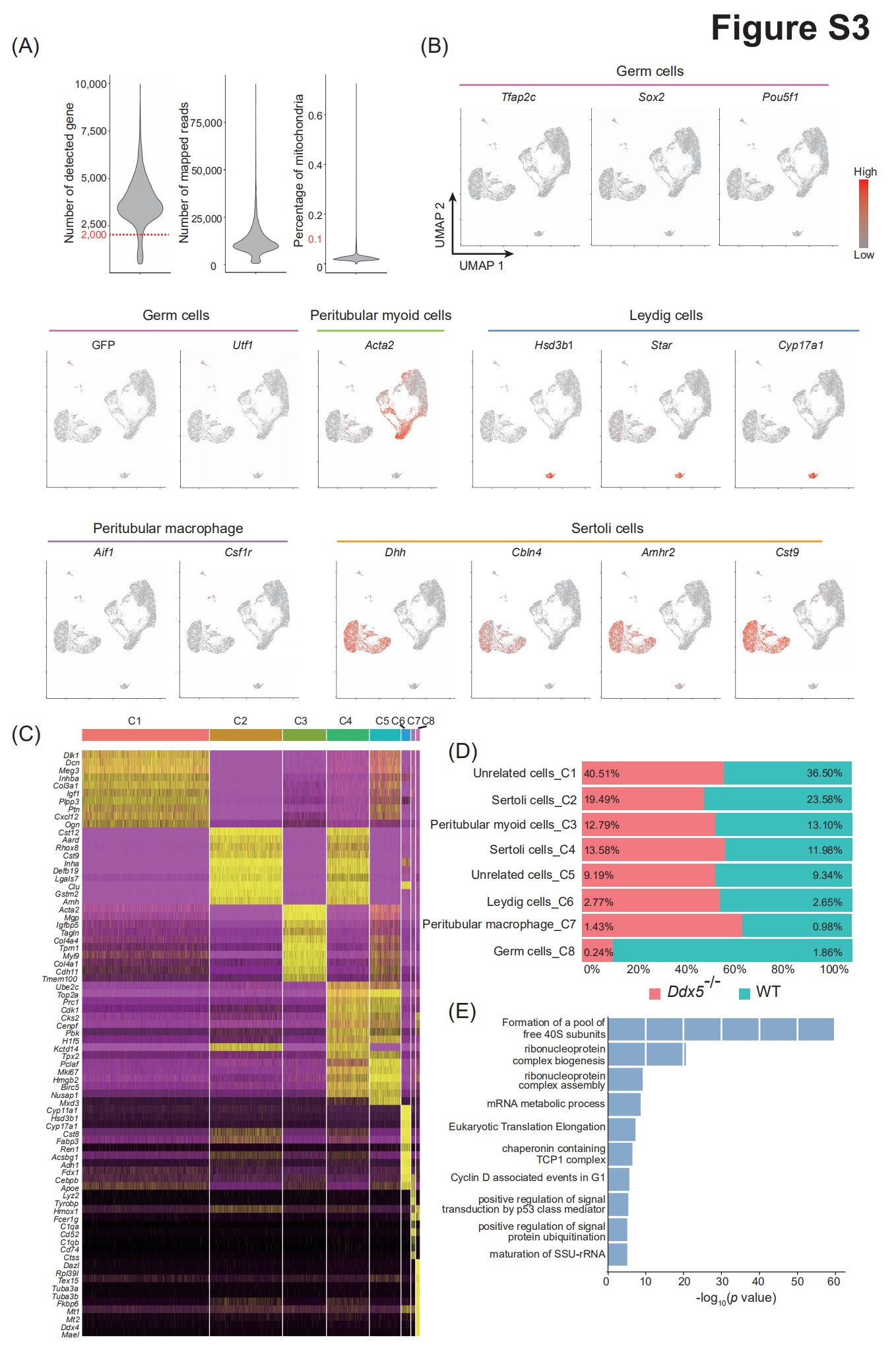

Supplement: Supplementary file 3 — Fig S3 [file CPR-54-e13000-s003.tif]
