# Supplementary material for: RNA helicase DDX5 acts as a critical regulator for survival of neonatal mouse gonocytes
Source: Cell Prolif. 2021 Mar 5;54(5):e13000. doi: 10.1111/cpr.13000 (PMC8088469; doi:10.1111/cpr.13000)
Supplement: Supplementary file 4 — Supplementary Material [file CPR-54-e13000-s005.docx]

**SUPPLEMENTARY METHODS**

**Mice**

The *Ddx5* floxed mice *Ddx5*^flox/flox^ were purchased from the European Mouse Mutant Archive (INFRAFRONTIER). *Mvh*-Cre and *Oct4*-GFP transgenic allele–carrying mice were purchased from the Jackson laboratory. All mice were maintained under specific-pathogen-free (SPF) condition in compliance with the guidelines of IACUC and the Guangzhou Institutes of Biomedicine and Health, Chinese Academy of Sciences (permit No. 2014015). Animals were individually housed under a 12 hr light/dark cycle and provided with food and water ad libitum. All efforts were made to minimize animal discomfort.

**Genotyping of mice**

Genotyping was performed by PCR using DNA isolated from tail tips. Forward primer (5’-GCTGCTCACATTGCCTTTGC-3’) and reverse primer (5’-CCTTCCCAACTGTCATTCTAAGG-3’) were used to detect wild-type allele (302 bp) and the floxed allele (420 bp). *Mvh*-Cre was genotyped with the forward primer (5’-CACGTGCAGCCGTTTAAGCCGCGT-3’) and reverse primer (5’-TTCCCATTCTAAACAACACCCTGAA-3’) to produce a 240 bp product. Wild-type and *Ddx5*^+/-^ male mice were used as the controls and homozygous *Ddx5*^-/-^ male mice were named as the knockout.

**Measurement of testicular weight and epididymal sperm count**

Testes weight and body weight from 12-week-old male mice were measured. The cauda epididymides of 12-week-old mice were minced in 1 mL PBS. After 30 min incubation at 37°C to allow the sperm to swim out, the suspension was then diluted and the number of sperms was counted with a hemocytometer.

**Fractionation of testicular cells**

Spermatogenic cells were fractionated by velocity sedimentation according to the method described previously.^1^ In brief, testes were dissected from mice immediately after euthanasia, and were rinsed and then cut into small pieces after the tunica albuginea was disrupted. Collagenase IV (Diamond, A004186-0001) and DNase I (NEB, M0303L) were then added at final concentrations of 2 mg/mL and 5 µg/mL, respectively, and testes were incubated in an incubator with 5% CO_2_ at 37°C for 15 min, with gently pipetting to accelerate the testes dissociation every 5 min. Then, the cell suspension was centrifuged at 1,000 rpm for 3 min and the pellet was digested with TrypLE™ Express Enzyme to dissociate the seminiferous tubules into single cells. Dispersed cells were collected by centrifugation at 1,000 rpm for 10 min. Cells were rinsed once and re-suspended with cold PBS. Dispersed cells were then laid on top of a prepared 2-4% BSA gradient and settled for 3 hr at room temperature. Fractions containing spermatocytes, round spermatids or elongating spermatids were collected manually and samples from each fraction were examined under microscope. Similar cell types were pooled together for western blotting.

**Flow cytometry**

Flow cytometry of testicular cells was conducted as previously reported.^2^.Briefly, testes were dissected from mice immediately after euthanasia, and were rinsed and then cut into small pieces after the tunica albuginea was disrupted. Collagenase IV and DNase I were then added at final concentrations of 2 mg/mL and 5 µg/mL, respectively, and testes were incubated in an incubator with 5% CO_2_ at 37°C for 15 min, with gently pipetting to accelerate dissociation of the testes every 5 min. Then, the cell suspension was centrifuged at 1,000 rpm for 3 min and the pellet was digested with TrypLE™ Express Enzyme to dissociate the seminiferous tubules into single cells. The separated testicular cells were re-suspended in Hanks’ Balanced Salt Solution (Thermo Fisher, 88284), containing 5% fetal bovine serum and 10 µg/mL Hoechst 33342 (Invitrogen, H1399), and incubated in an incubator with 5% CO_2_ at 32°C for 20 min. Propidium iodide (Beyotime, ST511) was added at 2 µg/mL. Cells were filtered through a 70 µm cell strainer (Corning, 431751) after brief incubation. Flow cytometry was carried out on a flow cytometer (BD LSRFortessa SORP, BD Biosciences).

**Western blotting**

Protein extracts from testes were obtained with cell lysis buffer (50 mM Tris-HCl (pH 7.6), 1% Triton X-100, 1 mM EDTA (Sigma-Aldrich, 20-158), 10% glycerol (Macklin, G810575), 1 mM dithiothreitol (DTT) (Sigma-Aldrich, 20-265), 1 mM phenylmethylsulfonyl fluoride (PMSF) (Merck, 52332) and protease inhibitor cocktail (Bimake, B14001)). Equal amounts of total proteins were applied to SDS-PAGE and transferred to polyvinylidene fluoride (PVDF) membrane (Bio-Rad, 1620177). The membrane was incubated with the diluted primary antibody and secondary antibody. The antibodies used in this paper are listed in Table S2.

**Histological analysis**

Testes and cauda epididymides of 12-week-old mice were isolated and fixed with 4% PFA overnight at 4°C. Samples were embedded with paraffin, and sectioned (8 μm). Hematoxylin and eosin (H&E) staining was conducted with standard procedure. The sections were imaged with ABX51 microscope system (Olympus).

**TUNEL staining**

The apoptosis of gonocytes in testes at P0 and P2 was detected by TUNEL staining according to the manufacturer’s protocol. Briefly, testis sections were incubated in 0.5% Triton X-100 and TUNEL reaction mixture (Beyotime, C1089) for 1 hr at 37°C. Cell nuclei were stained with 0.1 mg/mL DAPI. Fluorescent images were captured with a confocal microscope (LSM800, Carl Zeiss) and further processed with ZEN-2012SP2-blue software.

**Data processing of whole tissues**

Gene expression matrices were generated using the CellRanger software (version 3.1.0, 10× Genomics). Custom marker genes of GFP and CRE were added to the reference genome with *cellranger mkref* and raw data were processed further in R (version 3.4.3). The following quality control steps were performed: (i) genes expressed by less than 2 cells were not considered; (ii) cells that expressed fewer than 2,000 genes (low quality) were excluded from further analysis; (iii) cells in which over 10% of unique molecular identifiers (UMIs) were derived from the mitochondrial genome were removed. After filter, there were 11,278 cells (6,328 from wild type and 4,950 from *Ddx5*^-/-^) retained for downstream analysis (initial cell number is 12,186). Seurat package (version 3.1.5)^3^ was used to perform single cell from whole tissues clustering analysis. Briefly, data normalization and scaling were performed using *SCTransform* function with default parameters. A shared-nearest-neighbours (SNN) graph was constructed using the first 15 principal components before clustering cells using *FindClusters* function with a resolution of 0.1 and default parameters. We performed differential expression analysis to define the genes that marked each cluster using *FindAllMarkers* function with settings min.pct = 0.1, logfc.threshold = 0.25, using the Wilcoxon test.

**Data processing of germ cells**

To investigate the regulatory mechanism of *Ddx5* in germ cells at cluster 8, cells with number of detected counts of GFP and CRE more than 2 and 1 were reserved for further analysis respectively, and expression levels were transformed to log_10_(TPM + 1) values (TPM, Transcript per million). There were 47 germ cells from WT and 7 germ cells from *Ddx5*^-/-^ mice after fliting. We identified highly variable genes using the Seurat *FindVariableFeatures* function (selection method = "mean.var.plot", mean lower threshold = 0.0125, mean higher threshold = 3, dispersion threshold = 0.5). Data matrix (54 cells with highly variable genes only) was then auto-scaled and summarized by principal component analysis (PCA) using the FactorMineR package. The R packages DESeq2 (version 1.28.1)^4^ was used to identify the genes differentially expressed between germ cells from WT and *Ddx5*^-/-^ mice. The significant genes were identified as the absolute value of log_2_(fold change) > 1 and at a false discovery rate < 0.01. We used Metascape^5^ (http://metascape.org) and R package clusterProfiler (version 3.12.0)^6^ to perform functional enrichment analysis with the differentially expressed genes of each cluster.

**Quantification and statistical analysis**

Data is presented as means ± SD unless otherwise indicated in the figure legends. Sample numbers and experimental repeats are indicated in the figure legends. Two samples Student’s *t*-tests (two-tailed) were performed in qRT-PCR. Due to limited sample size in Figure 2D,G, Figure 4C, Figure 6C,D and Figure S2B, the Wilcoxon sum rank tests were performed instead of student’s *t*-test. Significance levels are: **p* < 0.05; ***p* < 0.01; ****p* < 0.001.

**SUPPLEMENTARY REFERENCES**

1. Bellve AR. Purification, culture, and fractionation of spermatogenic cells. *Methods Enzymol* 1993;225:84-113.

2. Bastos H, Lassalle B, Chicheportiche A, et al. Flow cytometric characterization of viable meiotic and postmeiotic cells by Hoechst 33342 in mouse spermatogenesis. *Cytometry A* 2005;65:40-49.

3. Stuart T, Butler A, Hoffman P, et al. Comprehensive Integration of Single-Cell Data. *Cell* 2019;177:1888-1902 e1821.

4. Love MI, Huber W, Anders S. Moderated estimation of fold change and dispersion for RNA-seq data with DESeq2. *Genome biology* 2014;15:550.

5. Zhou Y, Zhou B, Pache L, et al. Metascape provides a biologist-oriented resource for the analysis of systems-level datasets. *Nat Commun* 2019;10:1523.

6. Yu G, Wang LG, Han Y, et al. clusterProfiler: an R package for comparing biological themes among gene clusters. *Omics : a journal of integrative biology* 2012;16:284-287.
